# Characterization of intestinal microbiota in normal weight and overweight Border Collie and Labrador Retriever dogs

**DOI:** 10.1038/s41598-022-13270-6

**Published:** 2022-06-02

**Authors:** Giada Morelli, Ilaria Patuzzi, Carmen Losasso, Antonia Ricci, Barbara Contiero, Igino Andrighetto, Rebecca Ricci

**Affiliations:** 1 Department of Animal Medicine, Production and Health, University of Padua, 35020 Legnaro, PD Italy; 2 Laboratory of Microbial Ecology and Genomics, Istituto Zooprofilattico Sperimentale delle Venezie, 35020 Legnaro, PD Italy; 3 Research and Development Division, EuBiome S.R.L., 35129 Padua, Italy

**Keywords:** Ecology, Genetics, Microbiology, Zoology, Biomarkers, Pathogenesis, Risk factors

## Abstract

Obesity in dogs is an emerging issue that affects canine health and well-being. Its development is ascribed to several factors, including genetic predisposition and dietary management, and recent evidence suggests that intestinal microbiota may be involved as well. Previous works have shown obesity to be linked to significant changes in gut microbiota composition in humans and mice, but only limited information is available on the role played by canine gut microbiota. The aim of this exploratory study was to investigate whether composition of canine faecal microbiota may be influenced by overweight condition and breed. All the enrolled companion dogs were young adults, intact, healthy, and fed commercial extruded pet food; none had received antibiotics, probiotics or immunosuppressant drugs in the previous six months. Labrador Retriever (LR) and Border Collie (BC) were chosen as reference breeds and Body Condition Score (BCS) on a 9-point scale as reference method for evaluating body fat. The faecal microbial communities of 15 lean (BCS 4–5/9; 7 LRs and 8 BCs) and 14 overweight (BCS > 5/9; 8 LRs and 6 BCs) family dogs were analysed using 16S rRNA gene sequencing. Moreover, for each dog, the daily intake of energy (kcal/d) and dietary macronutrients (g/d) were calculated according to an accurate feeding history collection. *Firmicutes* and *Bacteroidetes* resulted the predominant phyla (51.5 ± 10.0% and 33.4 ± 8.5%, respectively) in all dogs. Bioinformatic and statistical analysis revealed that no bacterial taxon differed significantly based on body condition, except for genus *Allisonella* (*p* < 0.05); BC gut microbiota was richer (*p* < 0.05) in bacteria belonging to phyla *Actinobacteria* (family *Coriobacteriaceae* in particular) and *Firmicutes* (*Allobaculum* and *Roseburia* genera). No remarkable differences were recorded either for diversity indices (i.e., alpha diversity, *p* > 0.10) or for divergence within the sample set (i.e., beta diversity, *p* > 0.05). PERMANOVA tests performed on single factors demonstrated the tendency of dietary protein to influence the recruited dogs’ microbiota beta-diversity at amplicon sequence variant level (*p* = 0.08). In conclusion, the faecal microbiota of dogs involved in this exploratory study showed no major variations based on body condition. However, our findings suggested that certain bacterial taxa previously acknowledged in obesity-related studies may be detected in dissimilar amounts depending on canine breed.

## Introduction

Obesity is recognised as a multifactorial nutritional disorder whose prevalence has reached disturbing levels in humans and family dogs in developed countries over the last decades^1–8^.

Excessive body weight constitutes a major healthcare issue in veterinary practice because overweight (OW) and obese (OB) dogs have been proven to be at greater risk of developing hormonal disturbances and diabetes, as well as orthopaedic and cardiorespiratory diseases, and even cancer^9–13^; this, in turn leads to a shorter life span and a decreased quality of life^2,14,15^.

In addition to diet and exercise, obesity-related research has uncovered a complicated network of elements that contribute to weight gain that includes several environmental factors^3,8^, genetic predisposition^16–18^, and gut microbiota (GM) composition.

The GM is a large and complex community of microorganisms that plays a crucial role in promoting and maintaining the host’s overall health^19,20^. In humans and animal models, the impact of GM on metabolic homeostasis has been subjected to considerable attention over the last few years, and numerous studies have suggested its involvement in dietary energy harvest^21^, fat metabolism and storage^22–24^, satiety regulation^25^, and systemic inflammation^26^. Certain alterations in GM composition have been consistently observed in OB individuals and linked to obesity and metabolic syndrome^20,27–29^, such as decreased microbial diversity, impaired *Firmicutes*/*Bacteroidetes* ratio, and the overgrowth of pathogenic bacteria (e.g. *Staphylococcus aureus*, *Enterobacteriaceae*) and short-chain fatty acid (SCFAs) producers (e.g. *Faecalibacterium prausnitzii*)^20,26,30^. There is still much debate on the distinctive features of obesity-related GM however, and the clinical implications of its deviations have yet to be fully understood. Nonetheless, despite the phylogenetic and metabolic similarities among species, it is still unclear whether such findings can be translated to canine obesity models as well^31,32^.

Even though the GM has become a major research topic also in veterinary medicine, its composition is still far from being fully dissected in canine species^33^. Not many studies have investigated the differences in the bacterial composition of GM in lean and obese dogs so far, and the results have been controversial for both research and client-owned animals^34–42^. Moreover, the effects of obesity may have been masked by many other factors that appear to deeply influence canine GM, such as diet^30,38,43–45^, age^46–49^, breed^50^, and metabolic disorders^36^.

Aimed at acquiring better understanding of the role played by canine GM in obesity predisposition and development, this study investigated the taxonomical composition of the faecal microbiome of lean and overweight companion dogs of two breeds, one of which known to be particularly obesity-prone (i.e., Labrador Retriever^18,51^). In addition, advanced bioinformatic tools were employed to assess the impact of dietary features on the possible differences in the dogs’ gut populations. In order to guarantee the reliability of the data gathered on the potential effects of breed and obesity on GM composition as in a 2 by 2 factorial design-like study, individuals were selected carefully to minimise the influence of other factors.

## Results

### Characteristics of canine participants

Samples from 29 adult NW (n = 15) and OW (n = 14) dogs were analysed in this study. The recruited dogs’ features are reported in Table 1.

**Table 1:** Demographics of the dogs enrolled in this study (n = 29).

	NW (n = 15)	OW (n = 14)	*p*-value
**(a)**			
Age, y (LSmeans ± SE)	2.3 ± 0.5	4.1 ± 0.5	0.01
Body weight, kg (LSmeans ± SE)	25.4 ± 2.2	26.7 ± 2.3	0.31
BCS, /9 (median and range)	5 (4–5)	7 (6–8)	< 0.0001
Sex (n)			0.87
Male	9	7	
Female	6	7	
Lifestyle (n)			0.35
Indoor	5	8	
Outdoor	10	6	

	LR (n = 15)	BC (n = 14)	*p*-value
**(b)**			
Age, y (LSmeans ± SE)	3.5 ± 0.5	2.9 ± 0.5	0.37
Body weight, kg (LSmeans ± SE)	34.4 ± 0.9	19.0 ± 0.9	< 0.0001
BCS, /9 (median and range)	6 (5–8)	5 (4–7)	0.08
Sex (n)			0.87
Male	9	7	
Female	6	7	
Lifestyle (n)			0.35
Indoor	5	8	
Outdoor	10	6	

Statistical analyses were carried out to compare dogs based on body condition (a) and breed (b).

BCS, body condition score; NW, lean dogs; OW, overweight dogs.

BCS, body condition score; BC, Border Collies; LR, Labrador Retrievers.

The median age in months was 32 (range 13–61 m) in NW dogs and 33 (range 13–87 m) in OW dogs; there was a significant difference in age distribution between the two groups. The median age in months was 33 (range 13–87 m) in LRs and 32 (range 13–71 m) in BCs; there was no significant difference in age distribution between the two groups.

The median body weight of the NW dogs was 20.0 kg (range 12.5–40.0 kg) and 28.2 kg (range 18.6–40.0 kg) for the OW dogs; dogs in the OW group were not significantly heavier than dogs in the NW group. The median body weight of the LR was 35.0 kg (range 27.4–40.0 kg) and 19.5 kg (range 12.5–21.8 kg) for the BC; LR dogs were significantly heavier than BC dogs. However, when BCS was estimated to account for size differences, there was no difference between the two breeds.

There was no inequality in the distribution of sexes by body condition or breed nor in the distribution of individuals living mostly indoors or outdoors.

All dogs were fed commercial maintenance diets at the time of the study. The features of the dog foods consumed are reported in Table 2; three products were excluded because such information was lacking from their labels or the owner could not provide the label. No significant divergences were recorded in the overall nutrient composition of kibbles consumed by NW dogs compared to those consumed by OW dogs, except for ME and lipids. The recruited BC were found to consume kibbles that were more energy-dense and richer in lipids than the LR. No difference was detected between BC and LR when comparing energy consumption to metabolic weight (104.4 ± 6.7 and 97.4 ± 6.7 kcal ME/kg^0.75^ for BCs and LRs, respectively; *p* = 0.47); interestingly however, NW dogs were estimated to consume more energy per kg of metabolic weight than OW dogs (120.0 ± 6.1 and 81.9 ± 7.2 kcal ME/kg^0.75^, respectively; *p* =  < 0.01). When comparing the lipid intake related to the metabolic weight, it was revealed that BCs consumed about 29% more fat than LRs (4.9 ± 1.6 and 3.8 ± 1.3 g/kg^0.75^ for BCs and LRs, respectively; *p* = 0.09), even though this did not reach statistical significance; on the other hand, NW dogs were estimated to consume significantly more fat per kg of metabolic weight than OW dogs did (5.0 ± 1.4 and 3.4 ± 1.0 g/kg^0.75^, respectively; *p* =  < 0.01).

**Table 2:** Characteristics of the dry foods (n = 26/29) consumed by the recruited dogs (lean, NW, n = 14; overweight, OW, n = 12; Labrador Retrievers, LR, n = 13; Border Collies, BC, n = 13).

	NW	OW	*p*-value	LR	BC	*p*-value
CP (g/Mcal)	74.8 ± 3.0	74.0 ± 3.2	0.86	72.7 ± 3.1	76.1 ± 3.1	0.45
EE (g/Mcal)	42.0 ± 2.1	39.2 ± 2.3	0.37	37.3 ± 2.2	43.9 ± 2.2	0.04
CF (g/Mcal)	9.0 ± 0.6	8.2 ± 0.7	0.33	9.0 ± 0.6	8.2 ± 0.6	0.33
Ash (g/Mcal)	20.1 ± 0.9	21.1 ± 1.0	0.45	21.1 ± 0.9	20.1 ± 0.9	0.46
NFE (g/Mcal)	108.9 ± 7.2	116.5 ± 7.8	0.48	122.4 ± 7.5	103.0 ± 7.5	0.08
ME (kcal/100 g)	359.6 ± 4.7	355.1 ± 5.0	0.52	350.6 ± 4.4	364.5 ± 4.4	0.04

Data (g/1000 kcal; kcal/100 g) are given as LSmeans ± SE.

CP, Crude protein; EE, ether extract; CF, crude fibre; NFE, Nitrogen-free extract.

Labels of the dry foods consumed by the enrolled dogs were examined to identify whether prebiotic ingredients (e.g., fructo-oligosaccharides, mannan-oligosaccharides, beet pulp, inulin, psyllium) were included in their formulations. Out of 25 ingredient lists retrieved, 23 products contained at least one prebiotic (Supplementary Table 1). Probiotics (i.e., *Enterococcus faecium*) were also listed in the labels of two products.

### Faecal microbiomes

After pre-processing steps, a total of 507,339 sequencing reads were retained for further analyses, with a median of 8954.5 reads per sample. A total of 1690 ASVs were found, accounting for 8 phyla, 14 classes, 19 orders, 41 families, and 58 genera clearly defined. The taxonomic reconstruction and distribution of the bacterial populations encompassed by all analysed samples is reported in Supplementary Table S2.

**Figure 1:**
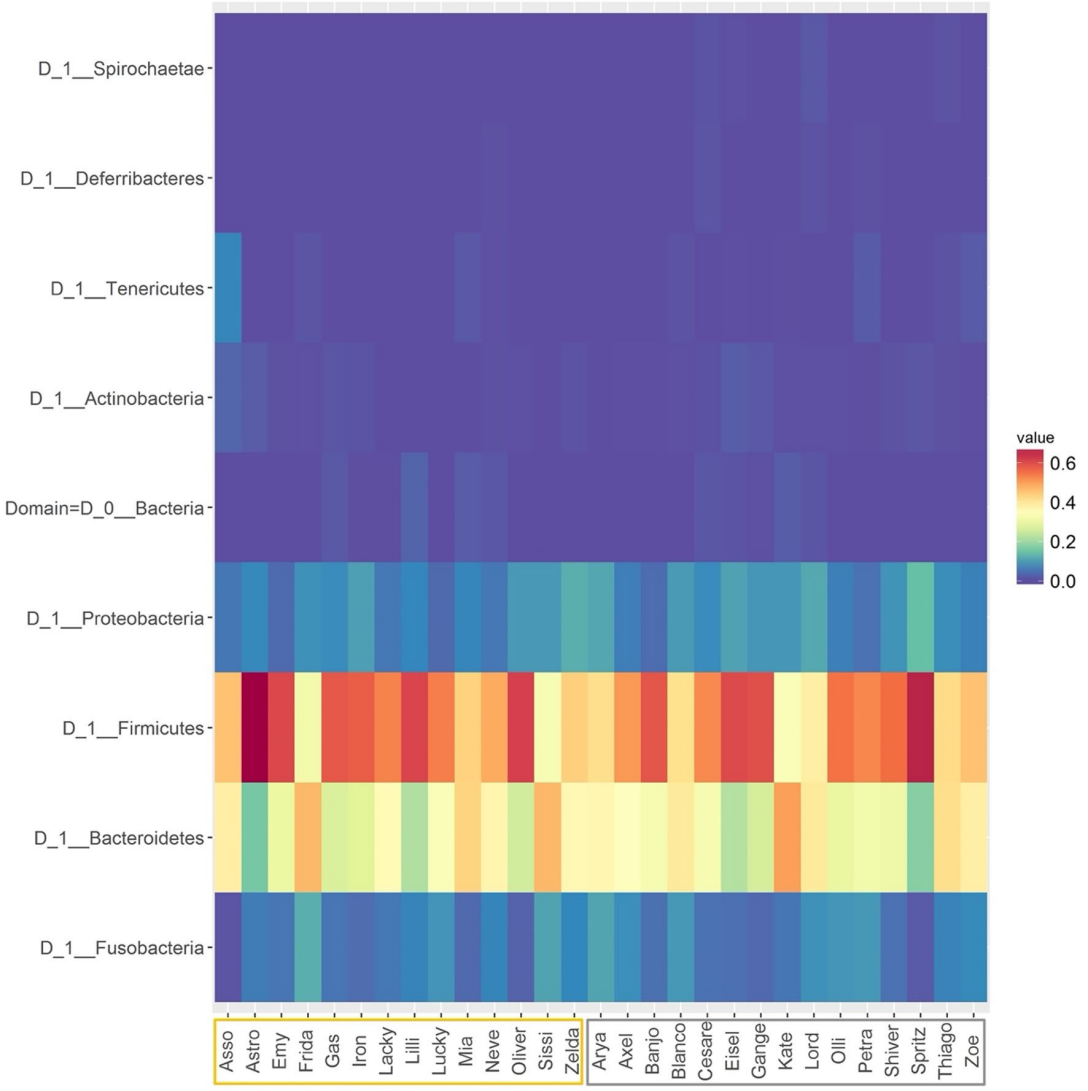
Heatmap displaying the relative abundance of predominant bacterial phyla in faecal samples belonging to the lean (grey box, n = 15) and overweight (yellow box, n = 14) canine populations, with individuals in rows and taxa in columns. Dogs’ names were used to distinguish the analysed samples. Colours represent relative abundances, the brighter the shade of red, the higher the abundance of the taxa. This figure was created using R version 3.4.3 (https://www.r-project.org).

Across all samples, the phyla which dominated the relative read abundances were *Firmicutes* (mean = 51.55, standard deviation = 9.99%) and *Bacteroidetes* (33.38 ± 8.54%), followed by *Proteobacteria* (7.59 ± 2.59%) and *Fusobacteria* (6.02 ± 2.70%) as shown in Fig. 1. Extensive variation was present among individual dogs at all levels, yet each taxon was tested for differential abundance among the sampled groups. Based on taxonomic proportions, the overall microbiome compositions of NW and OW dogs were not statistically different at any level (*p* > 0.05), except for genus *Allisonella* (0.00 ± 0.04% and 0.11 ± 0.04% in NW and OW, respectively; *p* < 0.05).

Certain significant differences in relative abundance of specific taxa emerged when LR were compared to BC (Table 3): the GM of BC was generally richer in bacteria belonging to phyla *Actinobacteria* (family *Coriobacteriaceae* in particular) and *Firmicutes* (*Allobaculum*, *Roseburia,* and other unknown genera). Based on these findings, a sample size of 27 animals per group is deemed necessary to detect significant differences in the proportion of genus *Allobaculum* between the two breeds; such sample size calculation was performed with 80% power and two-sided 5% level of significance.

**Table 3:** Faecal microbiome populations of Labrador Retrievers (LR, n = 15) and Border Collies (BC, n = 14) which differed significantly.

Taxa		LR	BC	*p*-value	*q*-value
Phylum	*Actinobacteria*	0.18 ± 0.15	0.80 ± 0.15	0.01	0.09
Class	*Coriobacteriia*	0.18 ± 0.12	0.64 ± 0.12	0.01	0.19
Order	*Coriobacteriales*	0.18 ± 0.12	0.64 ± 0.12	0.01	0.26
Family	*Coriobacteriaceae*	0.18 ± 0.12	0.64 ± 0.12	0.01	0.56
	*Porphyromonadaceae*	0.04 ± 0.09	0.32 ± 0.09	0.04	0.63
Genus	Unknown (fam. *Ruminococcaceae*)	0.74 ± 0.23	1.47 ± 0.23	0.04	0.67
	*Allobaculum*	0.36 ± 0.15	0.81 ± 0.15	0.04	0.67
	*Roseburia*	-0.01 ± 0.08	0.28 ± 0.08	0.02	0.67
	*Parabacteroides*	0.03 ± 0.08	0.29 ± 0.08	0.03	0.67

Data (% of sequences) are given as LSmeans ± SE.

**Figure 2:**
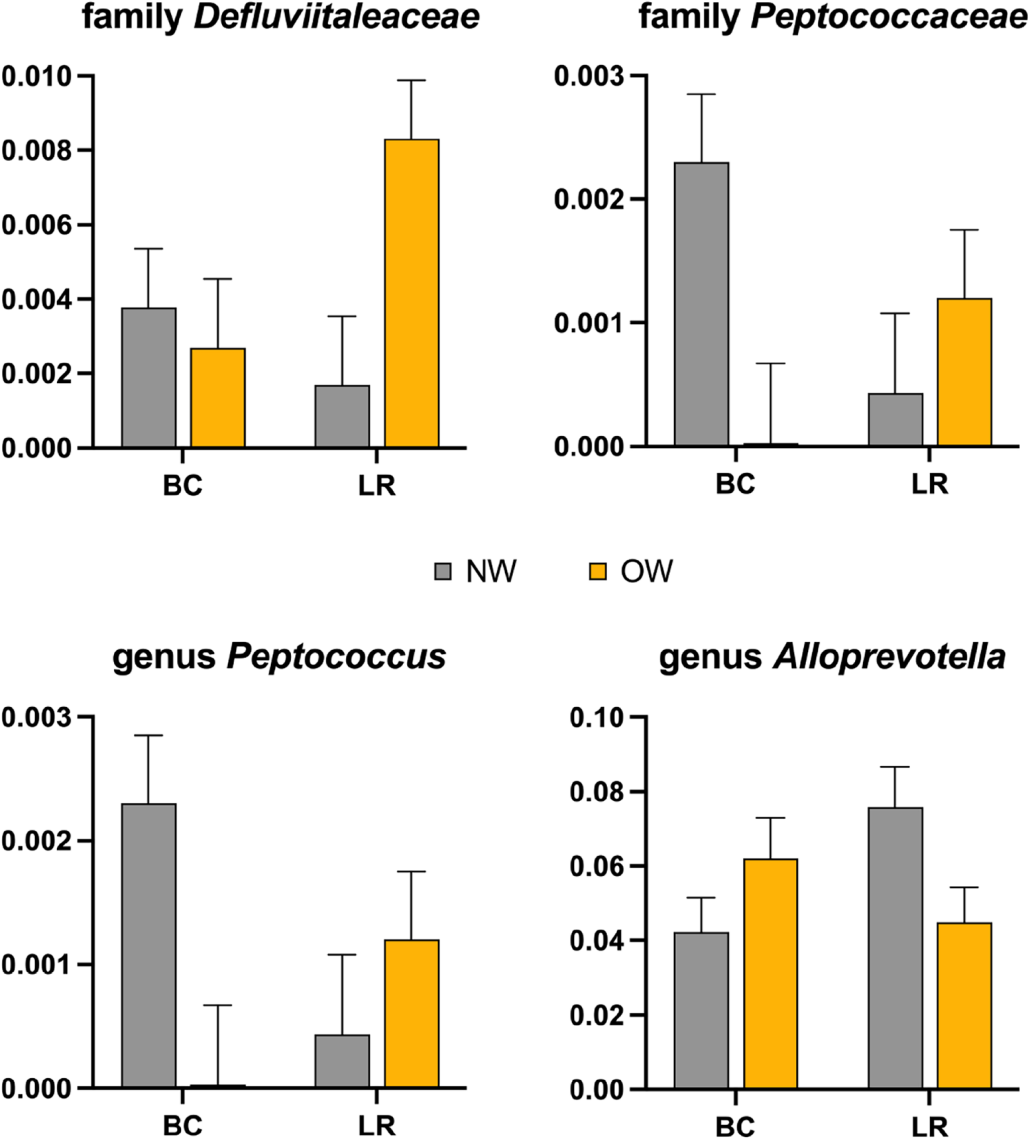
Significantly different bacterial taxa based on the interaction between body condition (lean, NW, n = 15; overweight, OW, n = 14) and breed (Labrador Retrievers, LR, n = 15; Border Collies, BC, n = 14) in the faecal microbiome of the recruited dogs. Data are presented as LSmeans and standard error of % of sequences. This figure was created using GraphPad Prism version 9.3.1 (https://www.graphpad.com/scientific-software/prism/).

There was a significant interaction in body condition by breed in GM composition, with NW-BC and OW-LR showing higher levels of families *Defluviitaleaceae* (*p* = 0.03) and *Peptococcaceae* (*p* = 0.02), as well as those of genus *Peptococcus* (*p* = 0.02); on the contrary, the same dogs showed lower levels of genus *Alloprevotella* (*p* = 0.02) (Fig. 2).

The following noteworthy differences were observed also between males and females (Table 4): female dog GM was generally richer in bacteria belonging to phyla *Fusobacteria* (order *Fusobacteriales* and genus *Fusobacterium* in particular) and *Firmicutes* (family *Peptostreptococcaceae* and genus *Turicibacter*); only *Collinsella*, a genus of *Actinobacteria*, was higher in male dog GM. Based on these results, a sample size of 15 animals per group is deemed necessary to detect significant differences in the proportion of phylum *Fusobacteria* between males and females; such sample size calculation was performed with 80% power and two-sided 5% level of significance.

**Table 4:** Significantly different faecal microbiome populations of male (M, n = 16) and female (F, n = 13) dogs.

Taxa		M	F	*p*-value	*q*-value
Phylum	*Firmicutes*	54.79 ± 2.46	46.86 ± 2.75	0.04	0.08
	*Fusobacteria*	4.82 ± 0.61	7.46 ± 0.69	< 0.01	0.17
Class	*Fusobacteriia*	4.82 ± 0.61	7.46 ± 0.69	< 0.01	0.15
Order	*Fusobacteriales*	4.82 ± 0.61	7.46 ± 0.69	< 0.01	0.20
Family	*Fusobacteriaceae*	1.62 ± 0.42	2.95 ± 0.46	< 0.05	0.55
Genus	*Fusobacterium*	1.62 ± 0.42	2.95 ± 0.46	< 0.05	0.60
	*Turicibacter*	0.24 ± 0.10	0.62 ± 0.11	0.02	0.60
	*Collinsella*	0.44 ± 0.08	0.16 ± 0.09	0.04	0.60

Data (% of sequences) are given as LSmeans ± SE.

In addition, at family level, the amount of *Defluviitaleaceae* differed conspicuously between dogs living indoors and outdoors (0.22 ± 0.13% and 0.61 ± 0.12%, respectively; *p* = 0.04); also, at genus level, the amount of *Butyricicoccus* differed slightly between dogs living indoors and outdoors (0.18 ± 0.12% and 0.52 ± 0.11%, respectively; *p* < 0.05), as well as *Escherichia-Shigella* (0.06 ± 0.26% and 0.81 ± 0.23%, respectively; *p* < 0.05).

Alpha diversity, in terms both of richness and evenness, showed no statistically significant difference between NW and OW dogs or between LR and BC (Kruskal–Wallis rank sum test, *p* > 0.05). Interestingly, at both the ASV and genus levels, microbial richness was lower in NW dogs, whereas equitability and uniformity (i.e., Pielou and Shannon indexes) were higher in the same group when compared to OW dogs (Table 5).

**Table 5:** Diversity indices (mean ± standard deviation) in the faecal microbiome of recruited lean (NW, n = 15) and overweight (OW, n = 14) dogs.

		NW	OW	*p*-value
ASV level (0% dissimilarity)	Richness (observed ASV)	849.00 ± 231.93	916.50 ± 151.97	0.36
	Pielou	0.76 ± 0.06	0.74 ± 0.05	0.46
	Shannon	5.11 ± 0.31	5.04 ± 0.32	0.60
Genus level (5% dissimilarity)	Richness (observed ASV)	65.60 ± 7.78	66.86 ± 8.87	0.66
	Pielou	0.66 ± 0.04	0.65 ± 0.05	0.93
	Shannon	2.76 ± 0.19	2.73 ± 0.22	0.86

**Figure 3:**
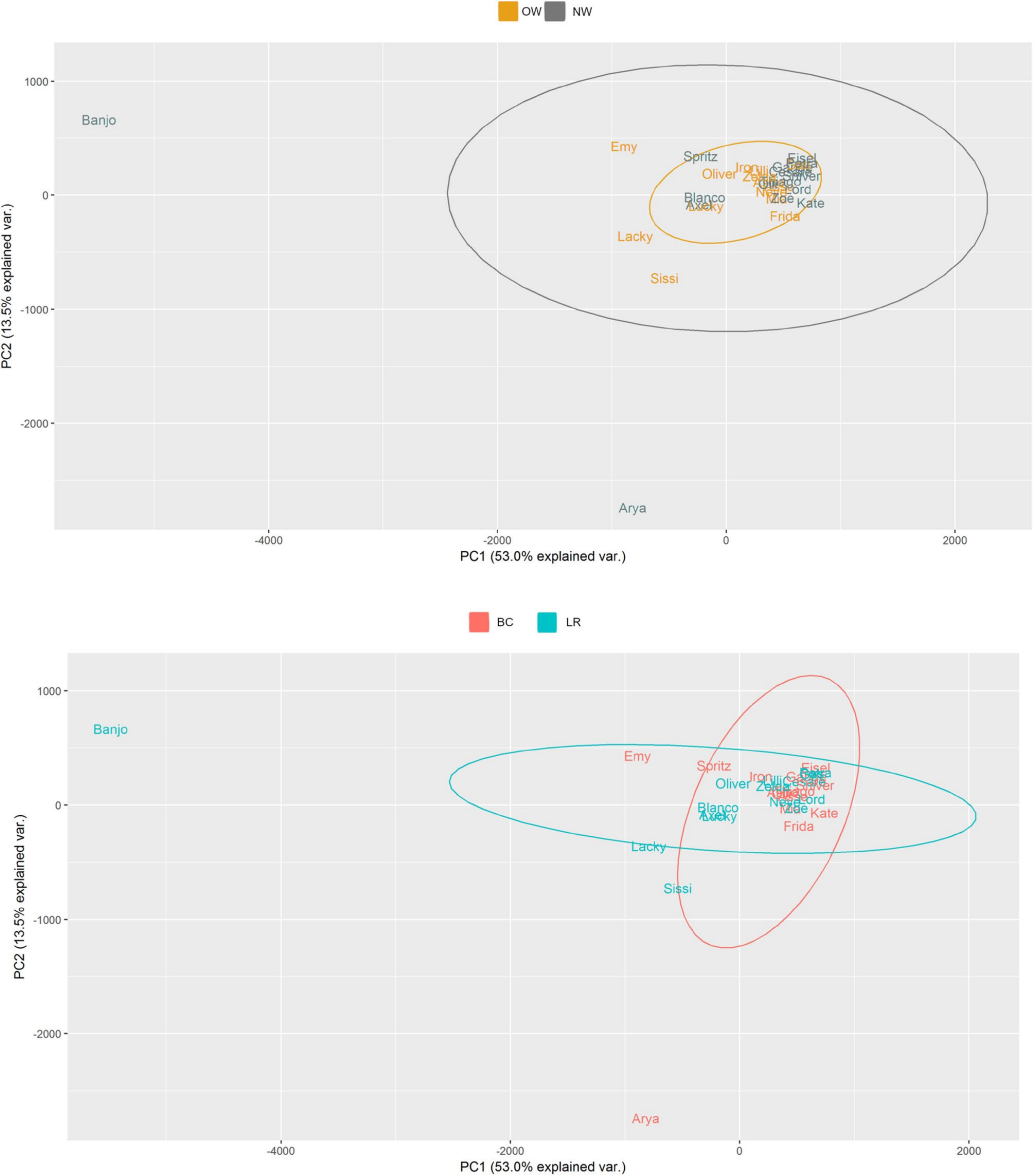
Principal component analysis (PCA) on Bray–Curtis dissimilarity at ASV; the colour scheme reflects (**a**) condition (lean, NW, n = 15; overweight, OW, n = 14) or (**b**) breed (Labrador Retrievers, LR, n = 15; Border Collies, BC, n = 14). Dogs’ names were used to distinguish the analysed samples. Neither plot showed a clustering between both groups. This figure was created using R version 3.4.3 (https://www.r-project.org).

**Figure 4:**
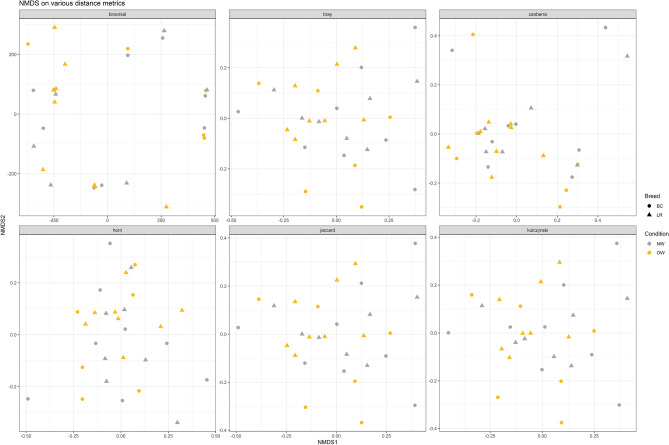
Non-metric multidimensional scaling (NMDS) plot based on six different beta measures generated at ASV level by condition (lean, NW, n = 15; overweight, OW, n = 14) and breed (Labrador Retrievers, LR, n = 15; Border Collies, BC, n = 14). Dots represent breed and colours indicate body condition. Dogs’ names were used to distinguish the analysed samples. No plot showed a clustering among groups. This figure was created using R version 3.4.3 (https://www.r-project.org).

**Figure 5:**
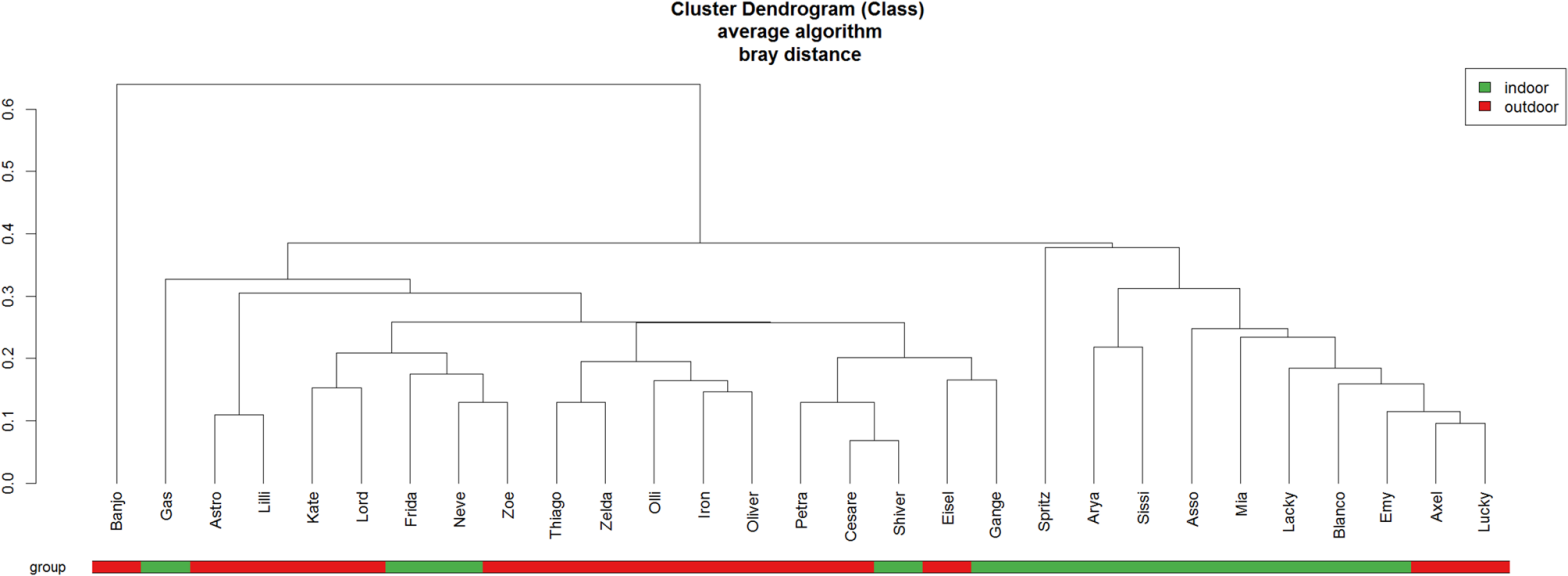
Cluster dendrogram of the average algorithm on Bray–Curtis distance between the recruited indoor (n = 13; green colour) and outdoor dogs (n = 16; red colour) at class level. Dogs’ names were used to distinguish the analysed samples. This figure was created using R version 3.4.3 (https://www.r-project.org).

All the analyses for beta diversity measures gave similar results, none of which displayed a differential clustering of microbial communities by host body condition or breed (Figs. 3, 4) at any level. Interestingly, two dogs (one NW BC and one NW LR) diverged conspicuously from the main grouping. A tendency to cluster was seen for lifestyle at class level (Fig. 5).

Lastly, the PERMANOVA test was performed to determine whether and to which extent host and diet factors modulated canine GM composition. None seemed to have a significant impact at any level, and only a tendency for proteins was detected in beta-diversity at ASV level (*p* = 0.08, R^2^ = 0.05477).

## Discussion

In this exploratory study, advanced bioinformatics analyses were performed to evaluate whether differences in canine faecal microbiota may depend on body condition and breed in a population of 29 client-owned dogs. The results did not reveal meaningful differences on the whole, suggesting that host unicity played a major role in modulating the dogs’ gut microbial communities. In other words, ASV that were peculiar for each analysed group could be mainly attributed to the individual contribution of one or few subjects.

The primary objective of the study was to identify the relationship between GM and the overweight condition in two dog breeds—one of which obesity-prone—using non-invasive methods. To this end, companion dogs were chosen to better reflect reality, not the artificial environment to which laboratory dogs are commonly subjected. Selection was nevertheless very strict in order to reduce the variables such as kinship, age, sex and reproductive status, health condition, and diet type that might engender excessive diversification.

No kinship among the recruited dogs was set as the first criterion in this study, due to the fact that the faecal microbiota of genetically-related dogs (i.e., puppies and their mothers, litter mates) have been shown to be more similar to each other than those of unrelated dogs^33,52^, and this could have led to biased sampling. Moreover, only the recruitment of one dog per household was allowed because also dog ownership seems to influence canine GM composition. In a study by Song and colleagues^53^, in fact, dog-owner pairs shared more microbial communities than non-household members.

As regards age, the greatest mutations in dog GM composition seemed to be linked to the stage of early development, specifically, the first weeks of age and weaning^20,46,52,54^. Results from previous studies suggest that canine GM composition changes also with ageing^46,47^. For the purpose of this study, it was reasonable to consider GM fixed at one year of age^52^, and since canine life stage classification is known to be affected by both the breed and size of dogs^55,56^, the upper age limit was fixed at 7 years, which falls into the category of young adult dogs in most studies.

The role of gender and spaying/neutering conditions have been scarcely considered when evaluating canine GM composition thus far, and dogs enrolled in previous works were mainly neutered. Particular attention was given to sexual status in the current study, as the enrolled individuals were intact, and males and females were equally represented. Such selection was made to eliminate neutering as a predisposing risk factor for obesity, given the fact that the interaction between gonadal steroids and body weight has been hypothesized as playing a role in the expression of many metabolism-related changes and food intake behaviours^57^. Also, the recruitment of intact subjects allowed us to see whether differences in the GM can be attributed to the influence of sex in the canine species. In contrast with the study by Mizukami et al.^47^ in which gender did not affect the analysis of the age-associated diversity in the GM of intact dogs, several significant differences were found in the current study, strengthening the assumption that sex hormones may play a role in shaping GM composition. In fact, although results have been inconsistent, sex-related differences have already been observed in the GM of mice and humans^44^ and some changes were not identified until puberty^44^.

Numerous diseases, both systemic and localized, influence or are influenced by GM, and are associated with dysbiotic states^58^. Medical interventions can also impact steady states in gut microbial communities, with the administration of antibiotics being the most deleterious^20^. In humans and laboratory animals, negative health events in childhood or growth phase (e.g., antibiotic use, malnutrition, premature birth) lead to abnormal GM development, and persistent disruptions in GM have been linked to multiple potential consequences that include inflammatory bowel disease, obesity, type II diabetes, and celiac disease. In particular, asthma, atopy, obesity, and autism spectrum disorder have been linked to childhood antibiotic use in humans^20^. Little is known about GM development in dogs or its disturbances in early stages. Short-term dysbiosis due to drug administration in adults is reversible and GM restoration occurs in few weeks. For this reason, the dogs recruited had not received any medical treatment during the previous six months; all dogs grew as healthy puppies, and 12 out of 29 had never received antibiotics in their lives.

It is no surprise that diet plays a major role in shaping canine GM composition; many studies, in fact, have shown that feeding dogs different foods resulted in distinct bacterial abundance and populations^43,59–61^. In this study, dogs fed entirely with commercial dry foods (kibbles, typically rich in carbohydrates) intended for maintenance were deemed eligible, whereas subjects fed home-prepared diets, mixed diets, conspicuous amounts of fresh food daily or prescription diets were excluded from the trial in order to achieve feed regimen consistency. In a study by Kim and colleagues^60^, in fact, dogs fed natural diets had higher GM diversity and more complex bacterial populations than commercial-fed groups regardless of differences in meat sources. Similarly, Mori and colleagues^61^ showed that different regimens exerted a significant effect on the GM of six healthy dogs fed each of the four commercially available prescription diets tested. Although statistical analyses revealed no remarkable differences in the dogs’ daily macronutrient intake (except for fat), the impact of the diet formulations on shaping the recruited dogs’ GM cannot be underestimated. Variations in the amount of protein and complex carbohydrates fed to pets can have a significant impact on their GM, and diets with different macronutrient ratios were shown to exert meaningful effects as well^38,58,62,63^. Compared to proteins and carbohydrates, much less is known regarding the role played by fat in influencing the GM^63,64^ and the dietary fat level was the only difference among the diets fed to the LR and BC dogs involved in this study. Also, most canine GM-centered studies have included animals fed with extruded diets that traditionally contain a high load of carbohydrates due to the inclusion of vegetable ingredients^58^. For canine diets however, it has been speculated that the kingdom of origin of the ingredients may be less important than the overall macronutrient composition. Extruded diets prepared exclusively with vegetable protein sources having similar macronutrient contents, in fact, did not significantly alter the microbiome of dogs when compared to traditional (i.e., mixed vegetable and animal) extruded diets^58,65^. Since as shown by PERMANOVA analysis the ecological indicators (i.e., alpha and beta diversity measures) and taxa proportions were not modified by the level of dietary macronutrients, the recruited dogs’ diets did not seem to contribute significantly to shaping the GM in this study, and this may support the hypothesis that selecting individuals eating exclusively maintenance kibble was effective in reducing confounding variables. As in previous studies^30,32,38,49,62,63,66^, the only tendency detected regarded proteins, which were shown to be one of the most influential dietary features in GM modulation in terms of both source of protein (quality) and ratio of protein to carbohydrate (quotient).

Prebiotic (i.e., selectively fermented ingredients that allow specific changes, both in the composition and/or activity in the GM that confer benefits upon host well-being and health) and probiotic (i.e., live organisms that confer beneficial effects on the recipient when delivered in adequate amounts) supplementation is known to induce significant modification of the GM^20,30,63^. Referred to as prebiotics, these include disaccharides (e.g., lactulose), polysaccharides (e.g., fructo-oligosaccharides), and nonstarch polysaccharides (e.g. inulin). Most kibble consumed by the dogs enrolled in this study were enriched with prebiotics, but their role in modulating the animals’ GM was difficult to discern. In fact, the ingredients on pet food labels must be listed in descending order by weight, inclusive of water weight, as per Regulation (EC) No 767/2009^67^. Also, the same European regulation does not force pet food manufacturers to report the total dietary fibre in the analytical constituent, as only the quantification of crude fibre is mandatory. This in turn did not allow the authors to know the precise amount of each prebiotic source included in the products, and the total prebiotic/soluble fibre amount could not be retrieved.

The degree of digestibility of a food determines the extent to which nutrients are digested and absorbed by the host; nutrients that escape digestion and absorption reach the colon and become thus available for microbial metabolism^63^. Therefore, it is reasonable to assume that the quality of dog foods (i.e., economic, premium, or super premium types of dog feeds) has a direct influence in terms of digestibility and GM composition. A recent study^68^ showed that feeding dogs with experimental diets having comparable nutrient concentrations but different nutrient digestibility was associated with changes in the composition of their faecal microbiota. Dogs enrolled in the current study consumed commercially available kibbles whose degree of digestibility was unknown; hence it was difficult for the authors to carry speculations over the impact of pet food quality on GM composition. Future research on the effect of diet on canine GM modulation should also take digestibility into account.

Overall, the dogs’ body condition did not seem to contribute markedly to shaping the faecal microbiota in the current study, a finding in agreement with the results obtained by Handl et al.^34^, Forster et al.^40^, and Macedo et al.^69^ Various shifts of diversity indexes have been recorded in other canine obesity-related studies as well^35,38,41,62^, in which dogs were however fed specific diets (e.g., high-protein diets, unequal protein to carbohydrate ratios) that exerted different effects on the GM of obese versus lean animals. Noteworthy variations in the relative frequency of certain bacterial groups were also seen in obese dogs after undergoing a weight loss program^39,41,69^_._ An in-depth inspection from phylum to genus level revealed that some taxa (1 phylum, 1 class, 4 families and 7 genera) were significantly affected by body condition, breed, or their interaction, but the real meaning of these findings can barely even be speculated.

Genus *Allisonella* was slightly more abundant in OW dogs, but to the authors’ best knowledge, no solid literature linking this taxon to canine, human or murine obesity and related disorders is currently available.

Curiously, compared to LR, BC showed a higher abundance of all significant taxa identified, most of which seem to be involved in obesity development. In previous canine obesity-related studies, an increase in phylum *Actinobacteria* and genus *Roseburia* was detected in obese family dogs^34^; an increase in phylum *Actinobacteria* has been associated with leaner dogs^35^ and dogs that underwent a weight loss program^69^ too, however. Higher proportions of *Allobaculum* have also been linked to both weight loss^41^ and excess body weight^62^; on the contrary, *Allobaculum* was more abundant in lean dogs than obese dogs or dogs that underwent weight loss in a recent study^69^. *Roseburia* is a SCFA-producing species believed to possess anti-inflammatory properties^70,71^, and it is interesting to note that human and murine studies have shown lower *Roseburia* spp. populations in obese individuals than in lean subjects, along with better efficiency by the GM in obese individuals in harvesting energy from the diet^72^. Similarly, several studies have shown the GM of obese mice to have lower abundances of *Allobaculum* spp., which has also been revealed to be a particularly active glucose utilizer in the body^73^. Both *Alloprevotella* and *Allobaculum* species are SCFA-producing bacteria that have been associated with improvements in obesity (e.g., decreased body weight, diminished low-grade inflammation) and insulin resistance^70,74^.

Surprisingly, faecal undefined *Ruminococcaceae* were also more abundant in BC. In the study by Kieler et al.^39^, *Ruminococcaceae* relative abundance was lower in dogs showing a faster weight loss rate; similarly, *Ruminococcaceae* count was highest in obese dogs and lowest in dogs that underwent weight loss in the study by Macedo et al.^69^ Given the role *Ruminococcaceae* plays in producing important amounts of acetic and propionic acids, the authors hypothesized that a GM that favours the production of short-chained fatty acids (SCFA) may negatively affect canine weight loss. Many butyrate-producers in the GM actually belong to the family *Ruminococcaceae*, whose higher concentration in obese mice potentially explains the higher caecal butyrate concentrations observed in the study by Garcia-Mazcorro et al.^75^. Another genus prevalent mostly in BC, *Parabacteroides*, has been hypothesized as playing a role in shifting the production of SCFA related to increased canine body weight^62^, yet recent studies demonstrated that some species belonging to genus Parabacteroides were able to alleviate obesity and obesity-related dysfunctions in mice, however. Supplementation with *Parabacteroides distasonis* decreased weight gain, hyperglycemia, and hepatic steatosis in obese mice^76^, while mice fed *Parabacteroides goldsteinii* showed reduced obesity rates and levels of inflammation and insulin resistance, as well as increased adipose tissue thermogenesis^77^.

Surprisingly, some clusterisation was observed in relation to the dogs’ lifestyle, namely: the time spent outdoors rather than indoors. Vilson and colleagues^52^ were the first to show that living environment affects dog GM, and that dogs living in big cities had higher GM diversity than dogs living in the countryside. The impact of the environment on GM composition and function is massive yet difficult to untangle due to the large number of variables. Although environmental extremes (e.g., altitude, temperature), pathogens, toxicants, pollutants, noise and physical activity have already provided evidence to this extent in humans and animals^78^, no data are available for canine species at present. In addition to diet, energy balance is sustained also by exercise, which influences the metabolism in a multitude of ways, and current evidence from animal models and humans shows that physical activity (or sedentary behaviour) and GM may interact in a complex relationship^78,79^.

Even though efforts were made to minimise the various confounding factors and recruit a homogeneous and representative canine sample, certain limitations in this study should be considered in order to better decipher its results. First of all, a higher number of samples could have improved the accuracy of the analyses conducted, but the decision to carefully select only clinically healthy, young adult, intact, kibble-fed dogs narrowed potential enrolment down inevitably. However, power calculations revealed that wide differences in the proportions of microbial taxa are needed to retrieve statistically significant results. Secondly, this study was conducted under field conditions: all dogs were client-owned and stools were collected outdoors; therefore, the role of domestic management and environmental conditions on individual GM composition should not be overlooked. Thirdly, the low-grade obesity of the recruited individuals in the OW group may have led to an underestimation of these findings. Finally, all dogs consumed dry foods of different brands; even if the study considered the impact of quantitative parameters of the diets consumed and found no significant differences between either breed or body condition status, the influence of qualitative peculiarities (e.g., protein sources, carbohydrate sources, inclusion of prebiotic ingredients) cannot be excluded.

## Methods

### Dog recruitment and metadata collection

A total of 29 canine stool samples were collected between December 2016 and January 2018. The samples were collected from privately-owned Labrador Retrievers (LR, n = 15) and Border Collies (BC, n = 14) whose owners enrolled in the study on a voluntary basis.

All dogs were recruited using the following inclusion criteria: dogs had to come from different households and be unrelated to one another; be young adults (i.e. 1–7 years old); be intact; be healthy (i.e. showing no clinical signs, no pathologies diagnosed) and not have received medications or taken antibiotics, probiotics or immunosuppressive drugs in the previous six months; have eaten a commercial dry dog food, and not have undergone a change in diet for at least four weeks prior to sample collection.

The nutritional status of the participating animals was checked by estimating the Body Condition Score (BCS) on a 9-point scale^80^ and then classified as lean (NW, BCS 4–5/9), overweight (OW, BCS 6–7/9) or obese (OB, BCS 8–9/9); every unit increase in BCS corresponded to an approximate 10% increase in body weight. Dogs were considered healthy (regardless of overweight or obesity status) if they had no past or recent history of severe illness and no abnormalities were identified on physical examination by a single veterinarian. Participating owners were asked to weigh their fasting animals using a scale the day before the scheduled appointment for sampling.

Along with signalment and medical history, information on dog lifestyle (i.e., physical exercise, indoor vs outdoor living) and dietary management was also collected from the owners. More specifically, quantitative and qualitative information on daily dietary intake was recorded, and the dog food’s analytical composition reported by the producers on labels was entered on a spreadsheet (Excel, Microsoft). The following data were recorded: product name; brand; moisture, when stated; crude protein (CP); ether extract (EE); crude fibre (CF); ash. Nitrogen-free extract (NFE) was calculated from label information (100% − moisture − CP − EE − CF − ash). For each product, metabolizable energy (ME, expressed as kcal/100 g) was calculated using the predictive equation for energy content based on the ‘modified Atwater’ factors of 3.5, 8.5 and 3.5 on as-fed basis for protein, fat and NFE, respectively^81^. A value of 8% moisture was assumed when moisture was not stated on the label as per European Regulation (EC) No 767/2009^67^. For each dog, the daily amount of energy and nutrients provided by the diet based on the average daily food intake (g/d) was calculated as kcal/d and g/1000 kcal/d, respectively.

### Faecal sample collection and DNA extraction

Stool samples were collected from family dogs during the scheduled appointments immediately after spontaneous defecation using a faecal swab (Fecal Swab™, Copan Diagnostics Inc., USA) inserted in the stool, being careful not to touch the soil, grass or surrounding items. Fresh samples were refrigerated at 4 °C and shipped to Laboratory of Microbial Ecology and Genomics at the Istituto Zooprofilattico Sperimentale delle Venezie (Legnaro, Padua) within 24 h, where they were processed as soon as received. Two total DNA extractions were performed by column-based kit QIAamp DNA Stool Mini (Qiagen, USA) for each sample in order to guarantee better representativeness of the entire microbial community. The extracted bacterial DNA was preserved at − 80 °C.

### Analysis of 16S rRNA sequences

Amplicons of V3-V4 regions of the 16S rDNA gene were sequenced on an Illumina MiSeq platform (LGC Genomics GmbH, Germany) using the bacterial primers described by Klindworth et al.^82^.

Data pre-processing was performed by using Quantitative Insights Into Microbial Ecology 2 (QIIME 2.0) pipeline (version 2017.12)^83^ and included quality filtering, merging of the paired-end fragments, chimera checking, and amplicon sequence variant (ASV) generation.

The obtained feature table was then analysed using an in-house implemented pipeline in the R environment (version 3.4.3, “Kite-Eating Tree”)^84^. The count table underwent normalization using scran package^85^ and zero-imputation by DrImpute tool^86^.

The final ASV table comprised 1690 ASVs in a total of 58 samples (29 stool samples with two technical replicates each). All reads were classified to the lowest possible taxonomic rank using QIIME2^83^ and a reference dataset from the SILVA database^87^.

#### Bioinformatics and Statistical analysis

##### Sample size calculation

Sample size calculation was performed based on 90% power and a type-I error of 5% in order to detect the effect of obesity on GM composition; the statistical program MedCalc v19.3.1 was used.

According to the findings of Salas-Mani et al.^41^ (in which six obese dogs were involved and underwent weight loss), a significant difference in the proportion of class *Clostridia* or genus *Allobaculum* between lean and obese subjects was expected if at least 2 animals per group were included. Based on the results by Handl et al.^34^ (who enrolled 22 lean dogs and 22 obese dogs), a significant difference in the proportion of phylum *Actinobacteria* or genus *Roseburia* between lean and obese subjects was expected if at least 124 and 37 subjects per group were included, respectively.

##### Dog features and metadata analysis

Due to the low number of OB subjects (2 dogs), OW and OB dogs merged into the same group (i.e., OW).

The metadata collected during recruitment were entered in a spreadsheet (Excel, Microsoft) and subjected to descriptive analysis. Categorical variables were evaluated using a two-proportion Z-test (i.e., sex and lifestyle) and Mann–Whitney test (i.e., BCS); continuous variables (i.e., age and weight) were evaluated for differences across groups using a one-way analysis of variance (ANOVA) with post-hoc Tukey’s pairwise comparisons. Differences in dog food macro-nutrient abundances and daily nutritional intake based on breed and body condition were assessed using a generalized linear model (GLM; SAS version 9.4). Differences were considered significant for a *p*-value of less than 0.05. In addition, *q*-values were calculated using the software R v4.0.5.

##### Microbiota profiling and diversity analysis

Statistical analysis of bacterial proportions was carried out using multi-factor ANOVA (SAS proc. GLM, version 9.4); *p*-value < 0.05 was considered significant. The model included the effects of breed, body condition, gender and lifestyle, and the interaction between breed and body condition.

Bioinformatic analyses were carried out using R (version 3.4.3)^84^ software packages and in-house scripts. The biodiversity of the samples (i.e., alpha-diversity) was characterized in terms of sample richness and evenness: the former was explored in terms of observed number of observed ASV (observed features), the latter was explored using the Pielou index; overall sample diversity was explored using the Shannon index through the *aindex* function from the DiversitySeq package^88^. The Kruskal–Wallis test was used to check for statistically significant differences in alpha-diversity metrics between groups (NW/OW dogs, LRs/BCs).

Similarity between samples (i.e., beta-diversity) was measured using several dissimilarity measures (i.e., Bray–Curtis, binomial, Canberra, Jaccard, Kulczynski, horn). The beta-diversity matrices obtained were used both for the hierarchical clustering of the samples and for dimensionality reduction analysis (Principal component analysis, PCA, and Non-metric Multidimensional Scaling, NMDS). PCA, NMDS and hierarchical clustering (based on Bray–Curtis distance) were used to investigate possible sample clustering by metadata factors.

Alpha- and beta-diversity analyses were performed at all taxonomic levels (ASV, Genus, Family, Order, Class, and Phylum); count tables for higher taxonomic levels were obtained by collapsing ASV abundances based on taxonomical assignation.

##### Dietary profiles and analysis of Variance using beta diversity distance matrix

Permutational Multivariate Analysis of Variance (PERMANOVA) based on the Bray–Curtis distance was computed at all taxonomic levels to assess which factors significantly contributed to shaping the variation of GM profiles using the *vegan* package^89^. All available continuous and categorical variables were considered: breed, BCS, gender, lifestyle, richness, evenness, and mean dietary energy, protein, lipid and carbohydrate intake.

Differences were considered significant for a *p*-value of less than 0.05 and trends were notified for *p*-value < 0.10.

### Ethical approval

All pet owners involved gave their informed consent for inclusion in the study by self-enrolling; anonymous information was collected as per General Data Protection Regulation (Regulation (EU) 2018/679). This observational study was carried out using non-invasive procedures on pets whose faecal matter was voluntarily donated by their owners; no ethics approval either within national or EU legal systems was needed for such procedure. All applicable international, national, and/or institutional guidelines for the care and use of animals were followed.

## Conclusion

In conclusion, the faecal microbial composition of lean and overweight dogs of two different breeds, one known to be obesity-prone, did not show major variations. Our results indicate that altered amounts of certain bacterial taxa previously considered in obesity-related studies conducted on dogs, humans, and rodents as well may be found in obesity-prone canine breeds. Also, this exploratory study suggested that sex and lifestyle may play a role in shaping canine gut microbiota. Further investigations involving a larger number of dogs of selected breeds are needed to investigate the role of body condition and breed on canine GM more deeply, as well as to uncover the influence of the microbial communities identified in canine obesity development.

## Supplementary Information

Supplementary Table S1.

Supplementary Table S2.

## Data Availability

The datasets generated and/or analysed during the current study are available in the Sequence Read Archive (SRA) repository; Accession: PRJNA813415. https://www.ncbi.nlm.nih.gov/bioproject/PRJNA813415.
